# Sex Differences in Body Ownership in Adults With Autism Spectrum Disorder

**DOI:** 10.3389/fpsyg.2019.00168

**Published:** 2019-02-04

**Authors:** Silvia Guerra, Andrea Spoto, Umberto Castiello, Valentina Parma

**Affiliations:** ^1^Dipartimento di Psicologia Generale, Università di Padova, Padova, Italy; ^2^Neuroscience Area, International School for Advanced Studies, Trieste, Italy; ^3^Department of Clinical Neuroscience, Karolinska Institutet, Stockholm, Sweden; ^4^William James Center for Research, ISPA – Instituto Universitário, Lisbon, Portugal

**Keywords:** autism spectrum disorders, body ownership, female phenotype, numbness illusion, sex differences

## Abstract

A strong male prevalence has been observed in autism spectrum disorder (ASD) since its definition, but the behavioral manifestations of sex disparity have yet to be clarified. Here, we investigate sex differences in the perception of the Numbness Illusion (NI), a procedure based on a tactile conflict, in adults with ASD and with typical development. We aim to assess if women and men with ASD perceive NI-dependent body ownership differently and whether sex differences emerge in individuals with typical development. To elicit the NI, participants pressed their right-hand palm against the confederate’s hand and stroked with the thumb and the index finger of their left hand the joined index fingers in a synchronous or asynchronous way. Results reveal that women with ASD present a reversed and atypical pattern for the NI compared to men with ASD and a group of matched controls. In particular, women with ASD report a stronger illusion than men with ASD, that is more evident in the asynchronous conditions. In the asynchronous condition, women in the ASD group report stronger NI as compared to women and men in the Control group, whereas men with ASD only to men in the Control group. In the typical sample, the NI emerges only in the synchronous condition and no sex difference is observed. We discuss our results in terms of potential advantage of women in sociality and sensory information processing that might lead women with ASD to use different modalities to solve the illusion compared to men with ASD. In sum, these outcomes describe sex differences in individuals with ASD in the domain of illusory perception. This may be used in the future to support the characterization of the female phenotype of autism.

## Introduction

Autism spectrum disorder (ASD) is defined as a heterogeneous disorder characterized by impairments in social interactions, communication, and repetitive and stereotyped behaviors, which is more commonly diagnosed in male than in female individuals (4M: 1F; Werling and Geschwind, 2013a,b; Halladay et al., 2015). The male prevalence in ASD is known from the origin of the disorder. Indeed, both Kanner (1943) and Asperger (1944) reported that the children with autism that they examined were exclusively boys. Consequently, the majority of the research on ASD has chiefly focused on male participants. As a consequence, the female phenotype of ASD is still poorly understood and research results in that area are highly inconsistent (Head et al., 2014). To date, these accounts have been used to explain the mechanisms underlying sex disparity in ASD with some studies suggesting that the female phenotype of ASD may be the result of innate characteristics that protect girls and women from ASD and make them less vulnerable to develop the core symptoms of the disorder (female protective effect or FPE; Robinson et al., 2013; Werling and Geschwind, 2013a). Other studies have advanced that female and male individuals are equally predisposed to develop ASD at the genetic level, but female individuals may have some factors - at the cognitive or/and neurobiological level – enabling them to better compensate for this risk during the lifespan (e.g., Skuse, 2007). Moreover, it has been proposed that sex differences in the development of the cognitive profile may lead to different manifestations of ASD in women and men (Carter et al., 2007). From the studies that have addressed sex disparity in ASD, it becomes evident that intellectual abilities play a role in facilitating the diagnosis of ASD in female individuals. In particular, in presence of intellectual disability, male and female individuals with ASD meet the diagnostic criteria in a similar way and the ratio of ASD diagnoses is 1F:1M; however, at high IQ scores, female individuals with ASD are underrepresented (4M:1F; Van Wijngaarden-Cremers et al., 2014). In this view, it has been supposed that high IQ scores may represent a confounding factor that leads to a missed diagnosis or a misdiagnosis in girls and women with ASD (Van Wijngaarden-Cremers et al., 2014).

In this connection, several studies based on clinical observations have suggested that high-functioning girls and women with ASD show different and less severe social and communication impairments compared to boys and men with ASD (Rivet and Matson, 2011; Werling and Geschwind, 2013a). In particular, girls and women with ASD tend to have better expressive behavior (e.g., sharing interest and/or reciprocal conversation; Lai et al., 2011; Head et al., 2014), less impaired social and communication skills (e.g., desire to interact with other individuals and/or better linguistic fluency; Carter et al., 2007) and different repetitive and stereotyped interest and/or activities (e.g., women’s interest tend to involve other people or animals rather than objects; Hiller et al., 2014; Lai et al., 2015) compared to boys and men with ASD. These greater social and communication abilities attributed as a feature of the female phenotype of ASD may help them to cope with social situations, masking some of the symptoms recognized as core symptoms of the male phenotype of ASD and causing misdiagnoses or late identification of ASD in girls and women (Wing, 1981; Attwood, 2007; Dworzynski et al., 2012; Hiller et al., 2016).

The above-mentioned evidence is in line with the extreme male brain theory (EMB; Baron-Cohen, 2002), which posits that the underlying sex disparity in ASD might be the ‘hyper-masculinization’ of some behaviors. In other words, the ASD profile may represent an extreme form of the typical male profile, which is characterized by enhanced systemizing and reduced empathizing skills. Deficits in empathy, in understanding and recognizing other people’s thoughts, perspectives and mental states [i.e., theory of mind (ToM)] are a trait frequently found in individuals with ASD (Baron-Cohen et al., 1985).

Developing functional motor, social and communication skills (Gallagher, 2000) and efficiently “walking in someone’s shoes” require the acquisition of the ability to differentiate the self from others and to compare the two entities. Such distinction can be achieved by developing a coherent sense of “bodily self,” which involves two distinct and interdependent aspects: agency and body ownership. Agency refers to the experience of generating and controlling actions and the events caused by them in the environment (Gallagher, 2000; David et al., 2008). Body ownership refers to “the feeling that my body belongs to me” (as in Stone et al., 2018), and to the fact that my body is different from other people’s bodies or external objects. The sense of body ownership, which origins from the integration of different sensory information (i.e., proprioceptive, tactile and visual stimuli) is present not only when we act, but also during passive movements (Van den Bos and Jeannerod, 2002) and it can be perturbed by the induction of illusions. Successful perturbation of body ownership has been achieved by presenting incongruent sensory stimulation able to shift the belonging of one body part to either external objects (e.g., a rubber object shaped like a human hand, Botvinick and Cohen, 1998; Ehrsson, 2007) or to another person’s body part (e.g., someone else’s finger, Dieguez et al., 2009; Martuzzi et al., 2015). The multisensory foundations of body ownership and its underpinnings have been usually investigated by means of the rubber hand illusion (RHI; Botvinick and Cohen, 1998). The RHI is an experimental paradigm that modulates the sense of body ownership by presenting incongruent sensory stimulations (i.e., looking at a rubber hand being stroked, while perceiving one’s unseen hand to be similarly touched), which generate a multisensory conflict that is solved by relocating the sense of feeling touched on one’s hand on the visible rubber hand. Several variants of RHI were developed to investigate the sense of body ownership: the virtual body illusion (Slater et al., 2008), the presentation of multiple hands (Folegatti et al., 2012) and the numbness illusion (NI; Dieguez et al., 2009; Martuzzi et al., 2015).

The latter is an experimental paradigm that allows for the manipulation of the experience of the body-ownership of fingers (Dieguez et al., 2009). In this paradigm, two individuals (i.e., the participant and a confederate) press the palm of their hands against each other. Then, both the participant and the confederate stroke with the thumb and the index finger of their respective free hand two joint index fingers in a synchronous (i.e., the two index fingers are stroked at the same time in up and down movement) or in an asynchronous way (i.e., one finger is stroked a time). In brief, stroking the fingers synchronously generates in the participants the sensation of owning the confederate’s finger as if it were his/her own finger. This illusion only emerges when the stroking occurs simultaneously. When the stimulation is asynchronous or performed by another person, the illusion is not perceived (or its illusory effects are reduced). Such illusory experience has been replicated in individuals with typical development (Dieguez et al., 2009; Martuzzi et al., 2015) and we have recently demonstrated its presence in adults with ASD (Guerra et al., 2017). However, whether body ownership illusory experiences are comparable among women and men with ASD is still unknown.

Here, we test whether women and men with ASD experience the NI in a similar way. Considering that women with ASD reportedly show less impairment in social information processing (e.g., Werling and Geschwind, 2013a), we foresee that the NI experience would be more efficient in women with ASD as compared to their male counterparts. In other words, we expect women with ASD to be more subjected to the NI, in virtue of a greater disruption of the sense of body ownership. If this were true, outcomes may point out for the first time to the existence of sex differences in the domain of sensory and illusory experiences in ASD and they may contribute to further support the characterization of the female phenotype of ASD.

To delineate whether the ASD diagnosis modulates the expression of body ownership and whether sex differences in the NI are evident irrespective of the ASD diagnosis, we additionally tested a Control sample of typical individuals. Indeed, evidence has suggested that sex influences many aspects of typical development (Kimura, 1992; Baron-Cohen et al., 2005). In particular, men score higher in spatial abilities (e.g., mental rotation tasks, map reading tasks; Kimura, 1999), while women exhibit better-than-male performance in social sensitivity, emotional recognition and verbal fluency tasks. Thus, considering the potential female advantage in the social domain and given that the development of adaptive social functioning requires an efficient sense of body ownership, we would expect a stronger disruption of the sense of body ownership during synchronous stroking in women with typical development compared to men.

## Materials and Methods

### Participants

The sample for the two experiments (also previously included in Guerra et al., 2017) consisted of 108 participants. Sample size for the group^∗^sex interaction^∗^conditions was estimated by means of the G^∗^Power 3.1 software (Faul et al., 2009) to have a power ≥ 95%, even in the case of a medium-small effect size (0.22). In Experiment 1, 39 adults with high-functioning ASD were enrolled (ASD; 29 M and 10 F; mean age ± 24.72; age range 19–31 years). In Experiment 2, 69 age- and FSIQ-matched control adults with typical development were recruited (43 M and 26 F; mean age ± 23.64; age range 19–33 years). Individuals with ASD were age-, gender- and full scale IQ-matched with the individuals with typical development (see Table 1). Full scale IQ was measured via the Wechsler Adult Intelligence Scale – Fourth edition (WAIS-IV; Wechsler, 2008; Italian language adaptation: Orsini and Pezzuti, 2013) or via the Wechsler Abbreviated Scale of Intelligence (WASI; Wechsler, 1999). Participants with ASD received a formal diagnosis from an expert, licensed clinical psychologist based on the Diagnostic and Statistical Manual of Mental Disorder – 5 (DSM-5) and the clinical evaluation was supported by meeting criteria on at least the Autism Diagnosis Observation Schedule (ADOS; Lord et al., 2000) or the Autism Diagnostic Interview – Revised (ADI-R; Lord et al., 1994; see Table 1), either on both. Participants with typical development had no history of ASD and they did not have any first or second-degree relatives with a diagnosis of ASD. Participants with ASD were recruited via the local Pediatric and Developmental Neuropsychiatric Clinics, while volunteers were recruited on campus at the University of Padova (Italy). The project was approved by the local ethical committee and the experimental procedures were in accordance with the Declaration of Helsinki (Williams, 2008). All participants signed a written informed consent prior to the beginning of their experimental session.

**Table 1:** Sample description.

	ASD Group				
	Males		Females	test	*p*-value
N	74% (*n* = 29)		26% (*n* = 10)	χ^2^(1) = 9.26	< 0.01^∗^
AGE	24.93 ( ± 3.33; range 19–31)		24.1 ( ± 3.60; range 19–30)	*t*_37_ = –0.66	0.509
Full scale IQ	113.33 ( ± 11.15; range 89–129)		114.14 ( ± 12.29; range 98–128)	*t*_37_ = 0.16	0.879
ADOS (total)	10.6 ( ± 5.41; range 3–23)		10.9 ( ± 4.61; range 6–22)	*t*_37_ = 0.16	0.871
ADI-R (total)	39.79 ( ±14.33; range 20–67)		35.1 ( ± 5.67; range 27–44)	*t*_37_ = –1.00	0.323

	**Control Group**				
	**Males**		**Females**	**test**	***p*-value**

N	62% (*n* = 43)		38% (*n* = 26)	χ^2^(1) = 4.19	< 0.05^∗^
AGE	24.14 ( ± 3.38; range 19–33)		22.73 ( ± 2.47; range 19–28)	*t*_67_ = –1.78	0.079
Full scale IQ	107.25 ( ± 12.28; range 92–121)		112.67 ( ± 4.50; range 108–117)	*t*_67_ = 0.72	0.487

		**ASD Group**	**Control Group**	**test**	***p*-value**

N	F	9% (*n* = 10)	24% (*n* = 26)	χ^2^(1) = 7.11	< 0.01^∗^
	M	27% (*n* = 29)	40% (*n* = 43)	χ^2^(1) = 2.72	0.099
	total	36% (*n* = 39)	64% (*n* = 69)	χ^2^(1) = 1.62	0.202
AGE	F	24.1 ( ± 3.60)	22.73 ( ± 2.47)	*t*_34_ = 1.25	0.220
	M	24.93 ( ± 3.33)	24.14 ( ± 3.38)	*t*_70_ = 0.98	0.330
	total	24.72 ( ± 3.37)	23.64 ( ± 3.10)	*t*_106_ = 1.68	0.095
Full scale IQ	F	114.14 ( ± 12.29)	112.67 ( ± 4.50)	*t*_34_ = 0.19	0.849
	M	113.33 ( ± 11.15)	107.25 ( ± 12.28)	*t*_70_ = 1.30	0.202
	total	113.5 ( ± 11.21)	108.7 ( ± 10.77)	*t*_106_ = 1.23	0.226


*ASD, autism spectrum disorder; IQ, intelligence quotient; ADOS, autism diagnosis observation schedules; ADI–R, autism diagnostic interview-revised. Age, IQ, ADOS and ADI-R refer to the mean, while standard deviation and range are given in parentheses. F, females; M, males; ^∗^*p* < 0.05.*

### Procedure

**FIGURE 1:**
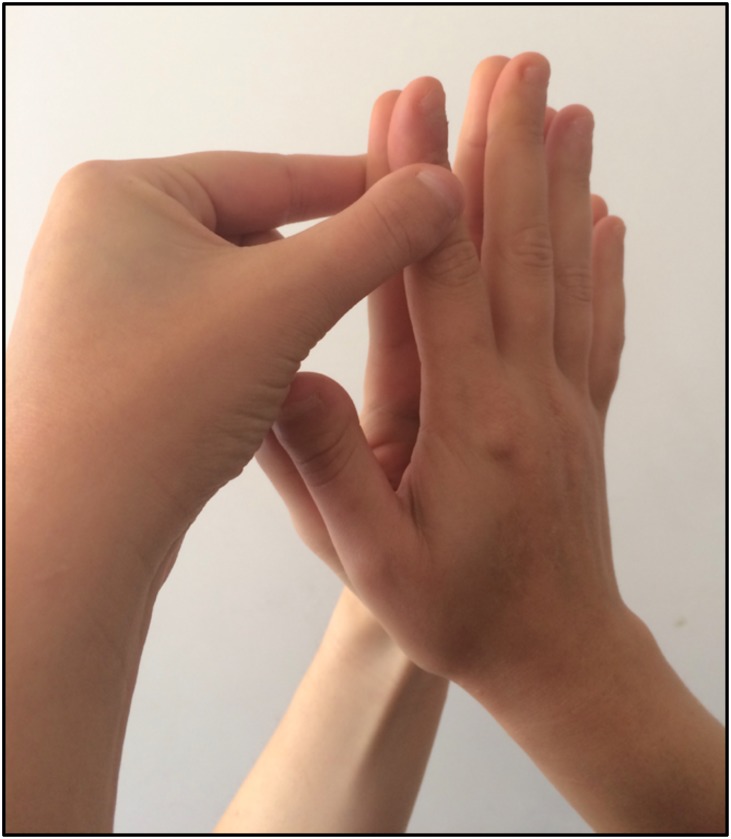
Procedure to induce the NI: the participant pressed the palm of his/her right hand against the left palm of the experimenter. In this posture, the participant stroked with the index and thumb of his/her free hand the joined index fingers (participant + experimenter). The stroking was performed synchronously (i.e., the joint index fingers were stroked at the same time) or asynchronously (i.e., the joint index fingers were stroked alternatively) by the participant.

The procedures were the same as in Guerra et al., 2017. Each participant and the experimenter sat facing each other. At the beginning of each trial, the participant was asked to press the palm of his/her right hand against the experimenter’s left-hand palm, which was lifted in the air (Figure 1).

In this posture, the participant stroked the dorsal side of the distal phalanges of the joined index fingers with the thumb and the index finger of the other hand, either in a synchronous or in an asynchronous way. Specifically, in synchronous conditions both the index finger and the thumb of the participant’s free hand started from the first phalanx and moved toward the third phalanx of the index finger of the receiver in a repetitive up-to-down movement. Instead, in asynchronous conditions the index finger of the agent started from the first phalanx, whereas the thumb started from the third phalanx of the index finger of the receiver and they moved in opposite directions stroking one finger at a time, alternatively. Before the beginning of the experimental phase, participants were trained to achieve a consistent stroking frequency and pressure. The frequency in stroking (i.e., 10 strokes in 10 s; 1 Hz) was constantly monitored by a co-experimenter by means of a timer to ensure that it was comparable across participants. The experimental design was a 2 × 2 factorial design. The factor Synchrony – how the joint index fingers were stroked – had two levels, namely synchronous (i.e., fingers stroked simultaneously) or asynchronous (i.e., fingers alternatively stroked). The factor Agent – who performed the stroking of the joint index fingers –had two levels, namely self (i.e., participant) and other (i.e., the experimenter). Given that the NI emerges only when the stimulation is self-administered and it primarily depends on the synchrony of the stimulation (i.e., Dieguez et al., 2009; Martuzzi et al., 2015; Guerra et al., 2017), the ‘Other’ condition has not been considered in this study (see Guerra et al., 2017 for an account on such condition in individuals with ASD). Indeed, the stroking performed by other people is not effective in inducing changes in the experience of the NI, irrespective of the type of synchrony of the stimulation. By removing this condition, we were able to gain power to evaluate the sex effects on the NI. This led to two experimental conditions, namely self-synchronous and self-asynchronous. Each condition was repeated four times in a pseudo-randomized order for a total of 8 trials. Each trial lasted 10 s. At the end of each trial, participants rated the strength of the illusion experienced during the task by means of a questionnaire composed by 5 questions presented on 5-point Likert scale (Dieguez et al., 2009; Martuzzi et al., 2015; Table 2). The scale ranged from 1 (completely disagree) to 5 (completely agree). In line with previous studies (e.g., Dieguez et al., 2009; Martuzzi et al., 2015), we considered scores higher than 3 indicating that a significant illusory experience was reported. Questions were repeated in a pseudo-randomized order across all trials, to reduce contextual influences on responses.

**Table 2:** Numbness illusion self-report.

*During the stroking of the fingers…*		

1. The felt sensation was strange				
2. I felt a sensation of numbness				
3. It seemed like my own stroked finger became wider in size				
4. It seemed like the experimenter’s finger became my own finger				
5. It seemed like I felt only the big finger was being touched				
Completely disagree	Disagree	Neutral	Agree	Completely agree
1	2	3	4	5


### Data Analysis

All statistical analyses were carried out with the R software (R package version 3.3.9; R Core Team, 2013) and, more specifically, by means of the *lme* function (nlme package version 3.1-131) to perform linear mixed effect models. For each participant, the mean of the responses across all conditions were computed to produce an individual index of the illusion experienced by the participant during the task (as in Dieguez et al., 2009 and Martuzzi et al., 2015). At first, the data from the ASD group were analyzed by means of fitting a linear mixed-effect model with Synchrony (synchronous and asynchronous) as within factor, Sex (women and men) as between factors. Then, data from the two groups (ASD and Control) were analyzed by means of fitting a linear mixed-effect model with Synchrony (synchronous and asynchronous) as within factor, while Sex (women and men) and Group (ASD and Control) as between factors. When significant interactions were retrieved, we conducted pairwise comparisons. The significance level was set at *p* < 0.05.

## Results

### Women With ASD Experience the NI More Strongly Than Men With ASD

**FIGURE 2:**
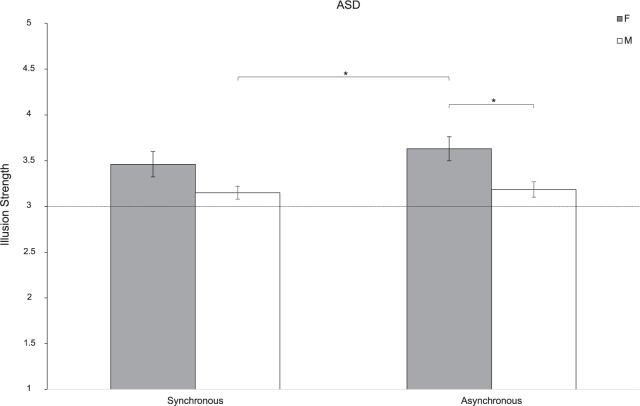
Strength of the NI for women (F) and men (M) of ASD group. Error bars indicate the standard error of the mean (SEM). ^∗^ = *p* < 0.05; The horizontal line with intercept 3 refers to the level at which the illusion was experienced by participants.

The analysis revealed that the NI was perceived differently by men and women with ASD [Sex: *F*(1,37) = 8.22; *p* = 0.007; partial-η^2^ = 0.182]. Indeed, the NI was perceived more clearly by women with ASD compared to men with ASD (*t*_37_ = 2.867; *p* = 0.007). Furthermore, women with ASD reported a greater disruption of finger’s ownership when the stroking was asynchronous than men with ASD both in the asynchronous (*t*_37_ = 2.885; *p* = 0.031) and the synchronous conditions (*t*_37_ = 3.108; *p* = 0.018). No main effect of Synchrony emerged [*F*(1,37) = 0.97; *p* = 0.331; partial-η^2^ = 0.042; Figure 2].

### Sex Affects the NI Experience in Individuals With ASD, but Not in the Control Group

**FIGURE 3:**
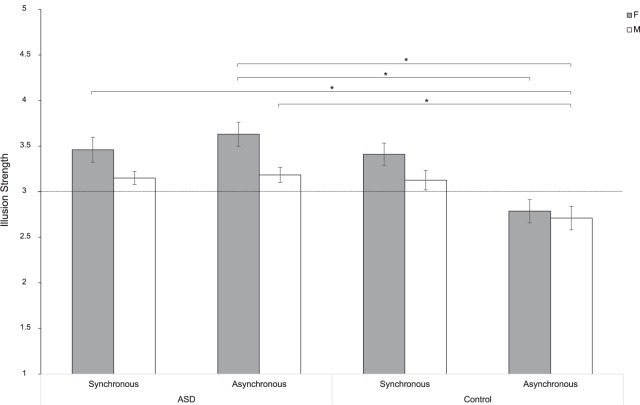
Strength of the NI for women (F) and men (M) in the ASD and Control groups in the synchronous and asynchronous conditions. ^∗^ = *p* < 0.05; Error bars refer to the standard error of the mean (SEM). The horizontal line with intercept 3 refers to the level of which the illusion was experienced by participants.

**FIGURE 4:**
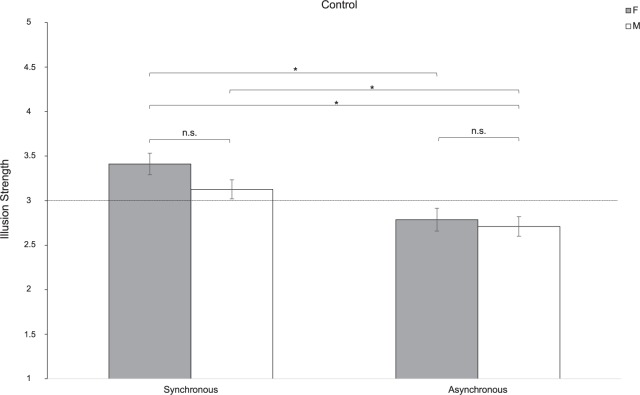
Strength of the NI for women (F) and men (M) in the Control group in the synchronous and asynchronous conditions. ^∗^ = *p* < 0.05; ns = lack of statistical significance. Error bars refer to the standard error of the mean (SEM). The horizontal line with intercept 3 refers to the level of which the illusion was experienced by participants.

To test whether this sex difference is characteristic of the ASD experience of the NI or it is also experienced by individuals with typical development, we fitted a linear mixed-effect model including the variable Group as a between factor. Results indicated a significant effect of Sex [*F*(1,104) = 4.79; *p* = 0.031; partial-η^2^ = 0.050]. Pairwise comparisons showed that women experienced the illusion more than men of both groups (*t*_104_ = 2.34; *p* = 0.021). More specifically, results indicated that women with ASD perceived a stronger illusion compared to women (*t*_104_ = 3.75; *p* = 0.007) and men (*t*_104_ = 4.34; *p* < 0.001) in the Control group, when the stroking was asynchronous. Furthermore, when women with ASD performed the stroking synchronously, results showed that the illusion was differently compared to men (*t*_104_ = 3.54; *p* = 0.014) in the Control group in the asynchronous condition. The same pattern was also observed in the comparison between men in the ASD and the Control group with respect to the asynchronous conditions (*t*_104_ = 3.27; *p* = 0.031). However, differently from what we found in the ASD group (see paragraph above), no significant sex differences were reported in the subjective experience of the illusion in the Control group [Group^∗^Sex: *F*(1,104) = 0.684; *p* = 0.410; partial-η^2^ = 0.007]. This suggests that women and men with typical development experience the NI in a similar manner (synchronous: *t*_104_ = 1.89; *p* = 0.555; asynchronous: *t*_104_ = 0.51; *p* = 0.999). These results were ascribable to the significant effect of Synchrony [*F*(1,104) = 30.91; *p* < 0.0001; partial-η^2^ = 0.104]. Indeed, the strength of the NI changed depending on the type of stroking (synchronous or asynchronous; Figure 3). This pattern holds true both for women (*t*_104_ = -5.85; *p* < 0.0001) and men (*t*_104_ = -5.01; *p* = 0.0001) with typical development and in the comparison between them (*t*_104_ = -4.67; *p* = 0.0002), but it is not evident in the ASD group (Figure 4). Indeed, the type of illusion perceived was different between the ASD and the Control group [Group: *F*(1,104) = 6.61; *p* = 0.012; partial-η^2^ = 0.075]. More specifically, the synchronous self-stroking produces the illusory effect of the NI in individuals of both the ASD and the Control groups. Such effect is not evident in Controls when the movement was performed asynchronously [Group^∗^Synchrony: *F*(1,104) = 26.77; *p* < 0.0001; partial-η^2^ = 0.204]. Average scores to each item separately per group, per gender and per condition are reported in Table 3, while a frequency table with respect of participants’ self-report responses are given in Supplementary Table S1.

**Table 3:** Self-report’s scores.

		ASD group		Control group	
		Males	Females	Males	Females
Synchronous	*Item 1*	3.38 ± 0.57	3.63 ± 0.44	2.99 ± 0.97	3.34 ± 0.94
	*Item 2*	3.08 ± 0.68	4.03 ± 0.36	2.60 ± 1.10	2.88 ± 0.99
	*Item 3*	3.07 ± 0.79	3.60 ± 0.49	3.02 ± 1.06	3.14 ± 0.97
	*Item 4*	3.26 ± 0.51	2.85 ± 1	3.38 ± 0.94	3.73 ± 0.77
	*Item 5*	2.97 ± 0.62	3.20 ± 1.21	3.64 ± 0.88	3.97 ± 0.73
		**3.15 ± 0.38**	**3.46 ± 0.44**	**3.13 ± 0.70**	**3.41 ± 0.62**
Asynchronous	*Item 1*	3.36 ± 0.71	3.43 ± 0.59	2.66 ± 0.93	2.83 ± 0.85
	*Item 2*	3.15 ± 0.63	3.90 ± 0.70	2.34 ± 0.96	2.60 ± 0.95
	*Item 3*	3.10 ± 0.81	3.93 ± 0.58	2.71 ± 1.02	2.47 ± 0.82
	*Item 4*	3.27 ± 0.69	3.33 ± 0.81	2.88 ± 0.77	2.99 ± 0.96
	*Item 5*	3.04 ± 0.76	3.58 ± 1.03	2.95 ± 0.91	3.05 ± 0.93
		**3.18 ± 0.45**	**3.63 ± 0.42**	**2.71 ± 0.72**	**2.79 ± 0.65**


*Average scores for each questionnaire’s item across all condition of both ASD and Control groups. Values refer to the mean and standard deviation of participants’ ratings to the self-reports’ questions in both synchronous and asynchronous conditions, respectively. See Table 2 for the description of each item. Values in bold type refer to the mean and standard deviation of the strength of illusion experienced by participants in different conditions.*

## Discussion

Researchers and clinicians are devoting more and more effort to understanding whether and how the differences between the female and male phenotypes of ASD can emerge. So far, the influence of sex differences in sensory experiences in ASD has only been marginally addressed. This study aimed to explore whether the experience of sensory-induced body ownership – measured behaviorally by means of the NI - differs between women and men with ASD. Our findings showed a clear sex difference in the strength of the NI experienced by individuals with ASD. Despite both women and men with ASD reported to experience the disruption of the body ownership over their own finger in the synchronous and in the asynchronous conditions, women reported to experience the illusion significantly more strongly than men.

To evaluate whether such sex difference in the experience of the NI is specific to ASD or it is a more general phenomenon, we also tested the effect of sex on the NI in a group of women and men with typical development. Comparing the performance of individuals with ASD with that of a group of matched controls, it emerges that women with ASD were more susceptible to the NI than women and men with typical development, especially when considering the asynchronous condition. This result acquires even more relevance when considering that no sex differences appeared when analyzing the Control group alone, showing that the NI manifests in a similar manner in both typically developing women and men. When focusing on the performance of men in both groups it is evident that both men in the ASD and Control groups were less susceptible to the NI compared to the women in both groups.

Two explanations, one focused on social skills and the other on sensory abilities, can be advanced to interpret these data. First, this finding may be interpreted as a reflection of the ability of women with ASD to better deal with socialization and empathy (e.g., Werling and Geschwind, 2013a). Indeed, the sense of body ownership has been deemed crucial in the development of adaptive social skills, particularly imitation and empathy (Gallese, 2003). The literature exploring the link between body ownership and empathy reveals that both participants with ASD (Cascio et al., 2012) and typical development (Farmer et al., 2012) who exhibited reduced empathetic skills were also those who were less susceptible to the RHI. Although not tested directly, we may speculate that the women with ASD in the present sample may present better empathic skills than their male counterparts. A second explanation, not in contrast with the previous one, may suggest that the increased susceptibility to the NI in women is the result of the ability of women to differentially focus on the sensory input received. Women seem to be more focused on the sensory information to solve the mismatch produced by the tactile conflict in the NI, whereas men rely less on such sensory information. This idea is in line with the evidence showing that girls with ASD score higher in the subscales of “Touch Response and Use” in the Tokyo version of the Childhood Autism Rating Scale (CARS) scale (Kumazaki et al., 2015) and that women with ASD report more sensory-motor symptoms than men with ASD (Moseley et al., 2018). Furthermore, increased sensory issues (e.g., noise hypersensitivity, unusual sensory interest, …) were reported more frequently in females with ASD than in males with ASD (Gould and Ashton-Smith, 2011; Lai et al., 2011). When directly comparing women with ASD and with typical development, self-reports suggest that they are both more sensitive to sensory stimulation than men (Tavassoli et al., 2014). This sensory perspective also fits with the evidence of a stronger NI’s experience in women in the synchronous (but not in the asynchronous) condition as compared to men. Indeed, results showed that the NI emerged in both groups when the stroking was synchronous, while, when the movement was performed asynchronously, only the participants in the ASD group experienced the illusion. This seems to suggest that the process differentiating between self and other in individuals with ASD is impaired, in that an excessive focalization on one’s own self can alter the perception of the self-other boundary (Noel et al., 2017).

An interesting observation, that was not part of our initial set of hypotheses, is related to the range of the responses given by the ASD group. Both women and men with ASD were highly reliable in providing the same rating of the strength of the NI. In other words, at the group level, the responses are locked around a limited range of options, as the inspection of the error bars suggests. This finding might be taken as evidence that the participants with ASD had difficulties in the understanding of the questions posed in the self-report. However, all participants with ASD presented a full-scale IQ comparable to that of controls, and this seems to be sufficient reason to believe that the instructions were understood and complied to the same extent as in the Control group. A more likely explanation for the reduced range of responses in the ASD sample can be found when interpreting this outcome in the context of the aberrant precision theory (Bolis et al., 2017). Such theory posits that individuals with ASD use abnormal strategies (i.e., perceptual hypersensitivity, hyper-attention to details,…) to make perceptual inferences. Such strategies, rather than maximizing the confidence in the sensory evidence estimated based on *a priori* beliefs (i.e., reducing the prediction error), tend to produce sub-optimal inferences about the nature of the sensory information. In other words, and compatibly with the neural instantiation of Bayesian inference from which this principle is extracted (e.g., Friston, 2005; Bastos et al., 2012), aberrant precision strategies emerge when the sensory bottom-up input and the top-down predictions about a stimulus are mismatching (i.e., the prediction error). In the context of the NI, the expectation of the participant is to feel their own index finger pressed against the hand of the experimenter as part of their own body. However, the sensory inputs (visual and tactile) produce an experience compatible with the reduction of body ownership for such finger (self) and attributing the ownership of that finger to the experimenter (other). Put in these words, it appears evident how the precision ascribed to the sensory evidence retrieved is imbalanced with respect to the *a priori* belief hold about the experience.

Despite the attention to details, including sensory ones in ASD (Martínez-Sanchis, 2014), one might also consider the lack of a significant difference between the synchronous and asynchronous conditions in ASD as the reflection of the lack in the perception of the NI and, instead, the evidence of greater sensory suggestibility in ASD. Although our data are not directly able to test this issue, we contend that this may be unlikely for at least two reasons. First, sensory suggestibility does not seem to be impaired in ASD. Specifically, when looking at sensory suggestibility in the RHI, it has been reported that the temperature of the hand subjected to the RHI similarly does not drop in participants with ASD and controls, calling for similar levels of sensory suggestibility across groups. Furthermore, studies on unusual tactile sensitivity in autism reported no differences in the domain of tactile perception across different tactile stimuli (e.g., detection of light touch, discrimination of the roughness of different sandpapers,…) when high-functioning individuals with ASD were compared with individuals with typical development (O’Riordan and Passetti, 2006; Cascio et al., 2008). However, these findings reflect a non-social aspect of sensory suggestibility and may not be impaired in ASD. In lack of other evidence directly linking socially relevant sensory suggestibility to the tactile domain, we turn to the evidence gathered from eye-witnesses. In this case, individuals with ASD are reported to be “no more or less suggestible than their typical counterparts” when directly asked to report about their experiences (Maras and Bowler, 2014). Second, if sensory suggestibility in the tactile domain is key to the perception of the NI, we should expect that the variability in sensory suggestibility would also be reflected at the level of the Control group. However, this is not the case, since a difference between the synchronous and asynchronous conditions is reported in the Control group, as expected when also looking at other illusory paradigms (Stone et al., 2018), but not in ASD.

In line with the call from the ASD and the scientific community for research into the female autistic phenotype (Halladay et al., 2015; Lai et al., 2015), the present study contributes to uncover of sex differences in adults with ASD in the field of the perception of body ownership by means of the NI, a novel procedure to better understand sensory and social issues in individuals with ASD. As for most innovative studies, some limitations in data interpretation exist and future studies will be needed to address them. First, although proportional to what found in the ASD population, the sample of women with ASD included in the present study is rather limited. Therefore, to confirm the present set of results we call for the replication of this study in a larger sample of individuals with ASD. Second, to better characterize the female phenotype of ASD a developmental perspective is needed. Indeed, testing our hypotheses from childhood to adulthood will allow to understand more deeply how sex differences in body ownership emerge over development. Third, to confirm the specificity of these results to ASD, it would be important to investigate sex differences in the NI in a group of individuals with non-ASD atypical development. Fourth, the administration of standardized self-reports on sensory perception (e.g., the sensory perception quotient; Tavassoli et al., 2014) and empathy (e.g., empathy quotient; Baron-Cohen and Wheelwright, 2004) can be used to probe the link between empathizing skills and the sense of body ownership during the NI. Indeed, testing the possible relationships between sex differences in the NI experience and individual empathic competences in typical and atypical populations might contribute to better understanding the processing underlying the behavioral sex disparity in ASD. Fifth, to further test whether the results hereby presented are confounded by sensory suggestibility, we suggest the inclusion of the sensory suggestibility scale (Gheorghiu et al., 1995) in future investigations.

To summarize, this is the first study exploring how women and men with ASD are affected by a sensory-induced illusion on the sense of body ownership. These results, discussed in the context of social and sensory issues typical of women with ASD, pave the way for the investigation of how sensory experiences can help define the female phenotype of ASD.

## Author Contributions

VP, UC, and SG conceived and designed the study. SG contributed to testing and data acquisition. AS, SG, and VP analyzed and interpreted the data. VP and SG drafted the manuscript. VP, UC, AS, and SG reviewed and edited the manuscript. All authors approved the final version of the manuscript for submission.

## Conflict of Interest Statement

The authors declare that the research was conducted in the absence of any commercial or financial relationships that could be construed as a potential conflict of interest.
