# Supplementary material for: Sex Differences in Body Ownership in Adults With Autism Spectrum Disorder
Source: Front Psychol. 2019 Feb 4;10:168. doi: 10.3389/fpsyg.2019.00168 (PMC6369199; doi:10.3389/fpsyg.2019.00168)
Supplement: Supplementary file 1 [file Table_1.DOCX]

Supplementary Material

**Sex Differences in Body Ownership in Adults with Autism Spectrum Disorder**

**Silvia Guerra^1^, Andrea Spoto^1^, Umberto Castiello^1^ and Valentina Parma^2,3,4^***

^1^ Dipartimento di Psicologia Generale, Università di Padova, Padova, Italy.

^2^ International School for Advanced Studies – SISSA, Neuroscience Area, Trieste, Italy.

^3^ Department of Clinical Neuroscience, Karolinska Institutet, Stockholm, Sweden.

^4^ William James Center for Research, ISPA - Instituto Universitário, Lisboa, Portugal

*** Correspondence:**

Valentina Parma

[vparma@sissa.it](mailto:vparma@sissa.it)

**Self-Report scores**

Participants were asked to rate the strength of illusion experienced after each trial (n=8) by means a specific self-report (see Table 2 in the main text). The self-report was composed by 5 questions presented on 5-point Likert scale, ranging from 1 (completely disagree) to 5 (completely agree), which were proposed in pseudo-randomize order across all trial following the procedure described in Dieguez et al., (2009) and Martuzzi et al., (2015). Then, ratings of all item across 8 trials were averaged to compute an individual index of the strength of the illusion experienced in different condition (i.e., synchronous and asynchronous). Below, a frequency table with the respect of the distribution of participants’ ratings in both experimental conditions (i.e., synchronous and asynchronous) across the 5 values of the Likert scale is provided.

**Table S1.** Frequency table of self-report’s scores

|  |  |  | ASD group | | Control group | |
| --- | --- | --- | --- | --- | --- | --- |
|  |  | Likert Scale | F | M | F | M |
| Synchronous | Item 1 | 1 | 0 | 1 (0.9%) | 9 (8.7%) | 24 (14%) |
|  |  | 2 | 5 (12.5%) | 22 (19.0%) | 12 (11.5%) | 32 (18.6%) |
|  |  | 3 | 15 (37.5%) | 37 (31.9%) | 28 (26.9%) | 49 (28.5%) |
|  |  | 4 | 10 (25%) | 44 (37.9%) | 45 (43.3%) | 55 (32%) |
|  |  | 5 | 10 (25%) | 12 (10.3%) | 10 (9.6%) | 12 (7%) |
|  | Item 2 | 1 | 1 (2.5%) | 9 (7.8%) | 14 (13.5%) | 43 (25%) |
|  |  | 2 | 2 (5%) | 29 (25%) | 27 (26.0%) | 48 (27.9%) |
|  |  | 3 | 8 (20%) | 38 (32.8%) | 25 (24.0%) | 27 (15.7%) |
|  |  | 4 | 13 (32.5%) | 24 (20.7%) | 34 (32.7%) | 43 (25%) |
|  |  | 5 | 16 (40%) | 16 (13.8%) | 4 (3.8%) | 11 (6.4%) |
|  | Item 3 | 1 | 1 (2.5%) | 12 (10.3%) | 5 (4.8%) | 24 (14%) |
|  |  | 2 | 3 (7.5%) | 25 (21.6%) | 29 (27.9%) | 39 (22.7%) |
|  |  | 3 | 15 (37.5%) | 36 (31%) | 24 (23.1%) | 26 (15.1%) |
|  |  | 4 | 13 (32.5%) | 29 (25%) | 38 (36.5%) | 75 (43.6%) |
|  |  | 5 | 8 (20%) | 14 (12.1%) | 8 (7.7%) | 8 (4.7%) |
|  | Item 4 | 1 | 6 (15%) | 8 (6.9%) | 0 | 13 (7.6%) |
|  |  | 2 | 13 (32.5%) | 15 (12.9%) | 15 (14.4%) | 28 (16.3%) |
|  |  | 3 | 8 (20%) | 44 (37.9%) | 15 (14.4%) | 32 (18.6%) |
|  |  | 4 | 7 (17.5%) | 37 (31.9%) | 57 (54.8%) | 79 (45.9%) |
|  |  | 5 | 6 (15%) | 12 (10.3%) | 17 (16.3%) | 20 (11.6%) |
|  | Item 5 | 1 | 8 (20%) | 10 (8.6%) | 1 (1.0%) | 8 (4.7%) |
|  |  | 2 | 8 (20%) | 28 (24.1%) | 9 (8.7%) | 18 (10.5%) |
|  |  | 3 | 5 (12.5%) | 45 (38.8%) | 6 (5.8%) | 31 (18%) |
|  |  | 4 | 6 (15%) | 22 (19%) | 64 (61.5%) | 86 (50%) |
|  |  | 5 | 13 (32.5%) | 11 (9.5%) | 24 (23.1%) | 29 (16.9%) |
| Asynchronous | Item 1 | 1 | 1 (2.5%) | 7 (6%) | 8 (7.7%) | 29 (16.9%) |
|  |  | 2 | 10 (25%) | 20 (17.2%) | 34 (32.7%) | 51 (29.7%) |
|  |  | 3 | 9 (22.5%) | 33 (28.4%) | 32 (30.8%) | 42 (24.4%) |
|  |  | 4 | 11 (27.5%) | 36 (31%) | 28 (26.9%) | 49 (28.5%) |
|  |  | 5 | 9 (22.5%) | 20 (17.2%) | 2 (1.9%) | 1 (0.6%) |
|  | Item 2 | 1 | 0 | 5 (4.3%) | 19 (18.3%) | 49 (28.5%) |
|  |  | 2 | 6 (15%) | 30 (25.9%) | 33 (31.7%) | 55 (32%) |
|  |  | 3 | 8 (20%) | 35 (30.2%) | 27 (26.0%) | 31 (18%) |
|  |  | 4 | 10 (25%) | 35 (30.2%) | 21 (20.2%) | 34 (19.8%) |
|  |  | 5 | 16 (40%) | 11 (9.5%) | 4 (3.8%) | 3 (1.7%) |
|  | Item 3 | 1 | 1 (2.5%) | 6 (5.2%) | 11 (10.6%) | 31 (18%) |
|  |  | 2 | 4 (10%) | 32 (27.6%) | 57 (54.8%) | 48 (27.9%) |
|  |  | 3 | 8 (20%) | 33 (28.4%) | 18 (17.3%) | 38 (22.1%) |
|  |  | 4 | 11 (27.5%) | 34 (29.3%) | 12 (11.5%) | 50 (29.1%) |
|  |  | 5 | 16 (40%) | 11 (9.5%) | 6 (5.8%) | 5 (2.9%) |
|  | Item 4 | 1 | 4 (10%) | 5 (4.3%) | 7 (6.7%) | 18 (10.5%) |
|  |  | 2 | 6 (15%) | 19 (16.4%) | 32 (30.8%) | 47 (27.3%) |
|  |  | 3 | 12 (30%) | 43 (37.1%) | 27 (26.0%) | 47 (27.3%) |
|  |  | 4 | 9 (22.5%) | 38 (32.8%) | 31 (29.8%) | 57 (33.1%) |
|  |  | 5 | 9 (22.5%) | 11 (9.5%) | 7 (6.7%) | 3 (1.7%) |
|  | Item 5 | 1 | 5 (12.5%) | 13 (11.2%) | 6 (5.8%) | 17 (9.9%) |
|  |  | 2 | 4 (10%) | 26 (22.4%) | 34 (32.7%) | 48 (27.9%) |
|  |  | 3 | 8 (20%) | 29 (25.0%) | 20 (19.2%) | 44 (25.6%) |
|  |  | 4 | 9 (22.5%) | 39 (33.6%) | 37 (35.6%) | 52 (30.2%) |
|  |  | 5 | 14 (35%) | 9 (7.8%) | 7 (6.7%) | 11 (6.4%) |

Note. Values refer to the frequency and the percent of the total of participants’ responses to each questions of the self-report in both synchronous and asynchronous conditions among the 5 values of the scale. See Table 2 in the main text for the list of self-report’s items.

**References**

Dieguez, S., Mercier, M. R., Newby, N. & Blanke, O. (2009). Feeling numbness for someone else’s finger. *Current Biology*, *19*(24), R1108-R1109. <http://dx.doi.org/10.1016/j.cub.2009.10.055>

Martuzzi, R., van der Zwaag, W., Dieguez, S., Serino, A., Gruetter, R., & Blanke, O. (2015). Distinct contributions of Brodmann areas 1 and 2 to body ownership. *Social Cognitive and Affective Neuroscience*, *10*(11), 1449-1459. <https://doi.org/10.1093/scan/nsv031>
